# Micro and nanoplastic inhalation during gestation disrupts placentation in the Sprague-Dawley rat

**DOI:** 10.21203/rs.3.rs-9861614/v1

**Published:** 2026-06-15

**Authors:** Chelsea M Cary, Nacala N Gadsden, Peihong Zhou, Laurie B Joseph, Calla Nguyen, Marianne Polunas, Michael J Goedken, Lauren M Aleksunes, Phoebe A Stapleton

**Affiliations:** Rutgers, The State University of New Jersey; Rutgers, The State University of New Jersey; Rutgers, The State University of New Jersey; Rutgers, The State University of New Jersey; Rutgers, The State University of New Jersey; Rutgers, The State University of New Jersey; Rutgers, The State University of New Jersey; Rutgers, The State University of New Jersey; Rutgers, The State University of New Jersey

## Abstract

Micro and nanoplastics (MNPs) are a ubiquitous environmental contaminant that humans are exposed through multiple routes. Multiple studies have demonstrated that MNPs deposit in human placental tissues and can translocate across the placental barrier. Maternal blood enters the placenta through uterine spiral arteries. During development of the placenta, trophoblasts enter the arteriolar lumen and invade the endothelial layer. This remodeling reduces vascular contractility and maintains maternal blood flow into the placenta. Simultaneously, the placenta increases surface area through angiogenic branching, facilitating the indirect contact of maternal and fetal blood spaces and promoting maternal-fetal exchange. To date, no groups have investigated how maternal MNP exposure affects these key steps of placentation. Therefore, in this study, pregnant Sprague Dawley rats were exposed to air containing polyamide-12 MNP throughout gestation. Placental morphology, invasion of spiral arteries, and angiogenic signaling were evaluated in male and female placentas at GD16 and GD20. Maternal MNP inhalation significantly reduced the relative distance of trophoblast invasion into the placental region that houses maternal spiral arteries. Additionally, MNP exposure increased staining of smooth muscle actin around maternal spiral arteries, indicating poor remodeling and likely reducing uteroplacental blood flow. Likewise, inhalation of MNPs altered the size and number of maternal and fetal blood spaces, favoring less surface area for maternal-fetal exchange. Lastly, significant changes in the expression and spatial distribution of angiogenic and antiangiogenic mRNAs that regulate vascular branching and surface area were observed. Future studies are needed to characterize the mechanisms by which polyamide-12 MNP influences placental hemodynamics.

## Introduction

The ever-rising production of plastic materials has led to widespread contamination of the environment by micro- and nanoplastics (MNPs). Similar to other constituents of particulate matter (PM), inhalation has been identified as a prominent route of human exposure to MNPs (Amato-Lourenço et al. 2021; Jenner et al. 2022; Yakovenko et al. 2025). The small size of MNPs facilitates their entry into the systemic circulation (Lee et al. 2024; Liu et al. 2024) and passage across selective biological barriers including the human placenta (Garcia et al. 2024; Ragusa et al. 2022a; Ragusa et al. 2021). Due to the detection of MNPs in the human placenta (Garcia et al. 2024; Ragusa et al. 2022a; Ragusa et al. 2021), breastmilk (Liu et al. 2023; Ragusa et al. 2022b), and meconium (Liu et al. 2023; Zhang et al. 2021), it is evident that MNPs interact with human maternal-fetal tissues throughout fetal and neonatal development. It has also been demonstrated that trophoblasts, the parenchymal cells of the placenta responsible for nutrient/waste exchange between the maternal and fetal circulation, internalize and transport MNPs (Dusza et al. 2022). Our laboratory has shown that MNPs deposit in the rodent placenta, are transported to fetal tissues after maternal exposure, and remain in offspring after birth (Cary et al. 2023a; Fournier et al. 2020; Moreno et al. 2024). Therefore, it is imperative to investigate the effects of gestational MNP exposure on *in vivo* placental function and development.

Humans and rodents possess a hemochorial placenta where the maternal and fetal circulations are separated by a thin barrier of syncytiotrophoblasts. The primary job of the placenta is to act as a conduit for nutrient and waste exchange between the maternal and fetal circulations. The efficiency of this exchange is directly proportional to morphological surface area. The placenta receives maternal blood following the migration of trophoblast into uterine spiral arteries. During pregnancy, these arteries are remodeled as invading trophoblasts replace maternal endothelium, leading to apoptosis of vascular smooth muscle cells and reduced vascular reactivity by trumpeting the terminal end of the spiral artery (Shukla and Soares 2022; Silva and Serakides 2016; Soares et al. 2012; Vercruysse et al. 2006). Spiral artery remodeling results in increased and sustained maternal blood into the placenta. Decreased spiral artery remodeling has been implicated in human pregnancy complications like preeclampsia and poor fetal outcomes (Dutta et al. 2018; Rana et al. 2019). Recent *in vitro* studies suggest that MNPs may influence trophoblast migration, however, whether maternal MNP inhalation has this effect *in vivo* is unknown (Wan et al. 2024b).

Human and rodent placentas are divided into functional zones identified as the decidua, basal plate/junctional zone, and intervillous space/labyrinth. The region of the uterus that is the site of blastocyst implantation is known as the decidua basalis or metrial gland in humans and rodents, respectively. While not technically part of the placenta, this maternal-derived tissue contains spiral arteries that supply placental tissue with maternal blood after early placental growth (Furukawa et al. 2019). In the rodent placenta, the decidua promotes maternal immune tolerance of the fetoplacental unit while the junctional zone participates in trophoblast differentiation and glycogen storage (Cline et al. 2014; Furukawa et al. 2019). The labyrinth zone represents the site of maternal-fetal exchange. Here, a complex network of intertwined maternal and fetal blood compartments facilitates exchange. In this region higher contact or surface area of the maternal and fetal blood spaces promotes increased exchange (Charest et al. 2018; Furukawa et al. 2014; Furukawa et al. 2019) and is achieved by angiogenic branching (Chen and Zheng 2014). As fetal metabolic demands increase throughout pregnancy, angiogenic signaling and branching modifies structure of the labyrinth, enabling increased maternal and fetal exchange. Induction of preeclamptic phenotypes in animals via reduced uterine perfusion or hypoxia result in disrupted placental morphology and function. This is evidenced by increased ratio of junctional zone surface area to labyrinth zone surface area, decreased expression of angiogenic factors, and increased expression of anti-angiogenic factors impaired, and fetal growth restriction (Gilbert et al. 2012; Natale et al. 2018; Umapathy et al. 2020; Wat et al. 2020). Similar outcomes have been identified in studies investigating the impact of maternal inhalation of PM_2.5_ and diesel exhaust on the placenta (Behlen et al. 2021; Chen and Yao 2017; Soto et al. 2017; Tao et al. 2022) Thus far, some studies have suggested that MNPs adversely impact trophoblasts by inducing cell death (Chen et al. 2025; Jia et al. 2025; Wan et al. 2024a; Wan et al. 2024b). However, the effects of maternal MNP inhalation on placental morphology and angiogenic signaling in the labyrinth have yet to be explored.

Given the detection of MNPs in placental tissues, it is imperative that the consequences of maternal MNP inhalation on spiral artery remodeling, placental morphology, and angiogenic signaling are characterized. Disruption of these characteristics may directly influence the ability of the placenta to support the fetus as described by rat models of placental insufficiency, culminating in fetal growth restriction and cardiometabolic deficits (Cary et al. 2025; Chou and Chen 2023; Dai et al. 2021; Sanders et al. 2004). Therefore, the aim of this study was to characterize how MNP inhalation throughout pregnancy influences uterine arterial remodeling and development of placental morphology in a rat model. We hypothesized that MNP exposure reduces spiral artery remodeling and decreases the size of maternal and fetal blood spaces in the placenta. Furthermore, fetal sex plays a major role in placental outcomes because trophoblasts are a fetal-derived cell type. Accordingly, we assessed placental vascular morphology at gestational days 16 and 20 in male and female rat placentas after repeated maternal MNP exposure throughout pregnancy.

## Methods

### Animals

Timed-pregnant Sprague-Dawley rats were ordered from Charles River Laboratories (Kingston, NY, USA) and delivered to a Rutgers University AAALAC-accredited vivarium. Normal chow and water were available to the pregnant dams *ad libitum*. Rats randomized into control and exposed groups and given a minimum of 24 hours to acclimate prior to aerosol exposure. Dams were selected for the collection of placental tissues on gestational days 16 or 20. Litter characteristics at gestational day (GD) 20 can be found in Supplemental Table 1. All experiments were humanely conducted in compliance with ARRIVE and NIH National Research Council guidelines with Rutgers University IACUC approval.

### Exposure

Pregnant dams were exposed to polyamide-12 particles (Orgasol ^®^ 2001 UD NAT 2, Arkema King of Prussia, PA, USA) at a concentration of 10.79 mg/m^3^ ± 0.96 mg/m^3^. In-house characterization of the naïve polyamide-12 physiochemical properties have been thoroughly established (Kidd et al. 2026; Moreno et al. 2026). Exposure began on GD 4 and continued 4 hours/day, 5 days/week through GD 15 or 19 in a whole-body inhalation facility (IEStechno, Morgantown, WV, USA) prior to tissue collection at GD 16 or 20. In brief, an acoustic generator connected to the air intake port of the inhalation chamber was used to aerosolize particles as previously described (Cary et al. 2023b; Moreno et al. 2024). Aerosol size characteristics within the inhalation chamber were quantified using a Scanning Mobility Particle Sizer (TSI Model 3080, Shoreview, MN, USA) and a High-Resolution Electrical Low-Pressure Impactor (Dekati HR-ELPI+, Kangasala, Finland) to assess electrical mobility and aerodynamic mobility, respectively. Characterization of the aerosolized polyamide-12 particles demonstrates the presence of particles within the nano- and micro- size range in the inhalation chamber (Supplemental Fig. 1). A target concentration of 10 mg/m^3^ was chosen based on the American Conference of Governmental Industrial Hygienists (ACGIH) guidelines for poorly soluble and non-cytotoxic inhalable aerosols, as described in the Safety Data Sheet provided by the manufacturer. This concentration is below the Occupational Safety and Health Administration (OSHA) guideline of 15 mg/m^3^ for inhalable dust. Pregnant rats were exposed to a concentration comparable to previously reported daily concentration assessments of MNPs in occupational settings (Antti-Poika et al. 1986; Eschenbacher et al. 1999).

### Histopathology

The right unit was externalized for harvest, fetuses were removed, and uteroplacental tissue was drop fixed in 10% neutral-buffered formalin until further processing. Fetal and placental sex were determined via qPCR of the Y-linked gene *Sry* and housekeeping gene *Gapdh* in fetal tissue or visualization of gonads at GD 16 or 20, respectively. Tissues were trimmed, processed, embedded, sectioned at 5 μm, Hematoxylin & Eosin (H&E) stained, by a single technician and examined by a board-certified veterinary pathologist via light microscopy. Sections were examined for the presence of overt pathologies, congestion, inflammation, apoptosis, and necrosis.

### Placental zone and vascular measurements

H&E-stained sections were scanned using light microscopy and assessed by a single technician (Axioscan 7/ Colibri 7 Carl Zeiss Instruments). Total placental area was quantified using a polygon measurement tool (Image-Pro Premier, Media Cybernetics). Area of maternal or fetal blood spaces and intrahemal barrier thickness were measured using a polygon and line tool, respectively (ZEN Blue Carl Zeiss Instruments). Measurements of maternal blood space area, fetal blood space area, and intrahemal barrier thickness were taken from the maternal-facing, midline, and fetal-facing regions of the labyrinth to limit selection bias of blood spaces. Maternal or fetal blood spaces within a 248 × 110 μm^2^ field were counted for each placenta (ZEN Blue, Carl Zeiss Instruments).

### Immunohistochemistry

Paraffin-embedded sections were deparaffinized and hydrated with xylene and sequentially decreasing concentrations of ethanol. Slides were boiled in a citrate antigen retrieval buffer for 20 minutes followed by three washes with ultrapure water. Endogenous peroxidases were quenched with Bloxall (Vector Laboratories CAT SP-6000–100) for 15 minutes. Tissue sections were washed with water and PBS, then blocked for 2 hours at room temperature with normal goat serum (Thermofisher Scientific CAT 50062Z). Tissue sections were incubated with α-smooth muscle actin primary antibody (SMA,1:1500 Abcam CAT ab5694) or control rabbit IgG for 15h at 4°C. After washes with PBS-T and PBS, tissue sections were incubated with biotinylated goat anti-rabbit secondary antibody (1:200 Vector Laboratories CAT BA-1000–1.5) for 30 min at room temperature. Avidin-biotin complex was applied to each section followed by washes with PBS-T and PBS (Vector Laboratories Vectastain Elite ABC-HRP Reagent, CAT PK-7100). Sections were then stained with 3,3′-diaminobenzidine peroxidase (Vector Laboratories ImmPACT DAB Substrate Kit, CAT SK-4105). Nuclei were counterstained with hematoxylin for 1 minute prior to dehydration and coverslip mounting. Slides were subsequently scanned using light microscopy (Carl Zeiss Instruments Axioscan 7/ Colibri 7). DAB/SMA staining was observed encircling blood vessels in the metrial gland, which corresponds to vascular smooth muscle of spiral arteries, was scored on a scale of 1 to 5 with a score of 1 having 0–20% circumscription of the vessel by DAB/SMA expression and 5 having 80–100% circumscription of the vessel by DAB/SMA expression. Relative area of staining in the labyrinth zone, which corresponds to placental pericytes, a vascular support cell (Garcia-Gonzalez et al. 2010; Harris et al. 2021; Li et al. 2021; Moreau et al. 2014; Nadeau and Charron 2014; Natale et al. 2020; Natale et al. 2018; Ohlsson et al. 1999; Singh et al. 2007; Zhou et al. 2021), was quantified using the Image-Pro Premier smart segmentation tool.

### Fluorescent in situ hybridization

Multiplex fluorescent localization of mRNA transcripts was carried out on 5 μm placental tissue sections according to the manufacturer’s protocol (Advanced Cell Diagnostics RNAscope^™^ Multiplex Fluorescent V2 Assay CAT 323270). Briefly, sections were baked for 1 hour and deparaffinized and incubated with hydrogen peroxide. Slides were boiled in target retrieval agent for 15 minutes and treated with a protease reagent for 30 minutes. Hybridization of probes to various mRNA transcripts (*Prl7b1*, *Tfpi, Vegfa*, *Kdr*, *Flt, Angpt1, Angpt2*, and *Tek*) was carried out for 2 hours at 40° C, followed by treatment with signal amplifiers. A horseradish peroxidase, fluorophore, and horseradish peroxidase blocker were applied to each slide in a manner corresponding to each probe channel. Erythrocyte autofluorescence was quenched by a 1.5-minute incubation of sections with Vector TrueVIEW (Vector Laboratories CAT SP-8400–15). Slides were then counterstained with DAPI, mounted, and scanned using fluorescence microscopy (Carl Zeiss Instruments Axioscan 7/ Colibri 7). Slide scan settings can be found in Supplemental Table 2.

### Statistics

Data were compared using two-way ANOVA and Sidak’s post hoc test or Student’s t-test where appropriate. The significance level was set at p < 0.05. 2-way ANOVA results of each compared endpoint are detailed in Supplemental Table 3. All data were analyzed using GraphPad Prism (GraphPad Software Inc., La Jolla, CA).

## Results

### Gross placental zones

**Figure 1:**
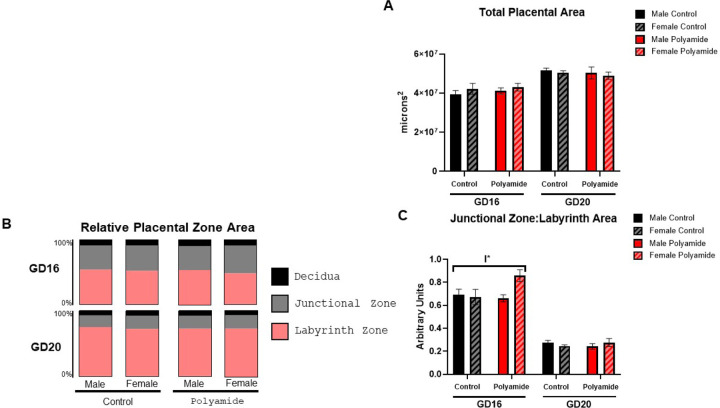
Regional placental morphology was measured in placentas at GD16 and GD20. Total placental area at GD16 and GD20 was quantified (A). A graphical representation of the relative placental zone areas at GD16 and GD20 is shown (B). Quantification of the ratio of junctional zone area to labyrinth zone area is presented (C). Data are presented as mean ± SEM. Significant exposure factor (p < 0.05) in the two-way ANOVA of age-matched placentas is denoted with E* and Ep-value when 0.05 < p < 0.1. Significant interaction effect between male and female placentas and control and exposed placentas at a given age (p < 0.05) are denoted with I* and I^p-value^ when 0.05 < p < 0.1, n= 7–11 dams/time point, n= 7–11 dams/time point/group.

Morphometrics of male and female control and exposed placentas were measured at GD16 and GD20. As expected, total placental area increased from GD16 to GD20. No significant treatment-related differences were identified for total placenta area in male and female offspring (Fig. 1A). A representative image with lines of demarcation for the placental zones can be found in Supplemental Fig. 2. Next, relative area of the decidua, junctional zone, and labyrinth zone were measured for male and female H&E-stained placental sections at GD16 and GD20, which also revealed no significant treatment-related differences (Fig. 1B). At GD16, the ratio of the junctional zone to labyrinth zone, a marker of placental dysfunction, was significantly increased in exposed female placentas, but not male placentas (Fig. 1C). No significant changes in this ratio were identified at GD20 (Fig. 1C). These data suggest that sex-specific changes in placental morphology after maternal MNP inhalation appear during GD16, a period of rapid placental growth.

### Residual smooth muscle actin

**Figure 2:**
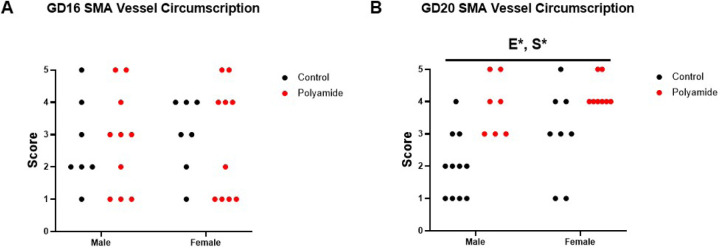
Graphical representations of scoring of α-SMA expression around vessels in the metrial glands of GD16 (A) and GD20 (B) placentas are shown. Significance was assessed via 2-way ANOVA with Sidak’s post-hoc test after transformation of data through ordered rank assignment. A graphical representation of these data demonstrates median score for each exposure group and sex. Significant exposure factor (p < 0.05) in the two-way ANOVA of age-matched placentas is denoted with E* and Ep-value when 0.05 < p < 0.1. Significant differences between male and female placentas at a given age (p < 0.05) are denoted with S* and S^p-value^ when 0.05 < p < 0.1. n=7–11 dams/time point/group.

Immunohistochemistry was conducted to visualize the localization of α-smooth muscle actin (α-SMA) in surrounding blood vessels in the metrial gland. α-SMA+ circumscription of the maternal vessels in the metrial gland was scored on a scale of 1 to 5 for GD16 and GD20 placentas. Representative images for each score can be found in Supplemental Fig. 3. At GD16, no change in α-SMA+ circumscription of vessels was detected between control and polyamide-exposed placentas (Fig. 2A). Raw scores are provided in Table 1. However, at GD20, MNP exposure resulted in an increased median score for α-SMA+ vessel circumscription (Table 1), independent of placental sex, as graphically represented in Fig. 2B. At GD20, sex was a significant factor in the outcome of α-SMA+ vessel circumscription, although there was no significant interaction effect. These data are indicative of impaired trophoblast remodeling of maternal vessels after MNP exposure.

### Metrial gland interstitial and endovascular trophoblasts

**Figure 3:**
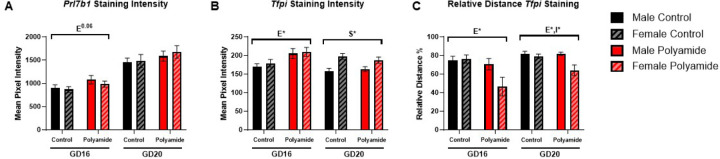
Localization of mRNA transcripts for *Prl7b1* and *Tfpi* were assessed in the metrial glands of GD16 and GD20 placentas to assess invasive and endovascular trophoblast invasion. Quantification of staining intensity and relative area stained was carried out for *Prl7b1* (A) and *Tfpi* (B) in the metrial gland. Relative distance of *Tfpi* staining into the metrial gland from the junctional zone was also assessed (C). Data are presented as mean ± SEM. Significant exposure factor (p < 0.05) in the two-way ANOVA of age-matched placentas is denoted with E* and Ep-value when 0.05 < p < 0.1. Significant interaction effect between male and female placentas and control and exposed placentas at a given age (p < 0.05) are denoted with I* and I^p-value^ when 0.05 < p < 0.1, n= 7–11 dams/time point/group.

Metrial glands of GD16 and GD20 placentas were stained for markers of invasive (*Prl 7b1*) or endovascular (*Tfpi*) trophoblasts. Representative images for each exposure group, age, and placental sex can be found in Supplemental Figs. 4 and 5 for *Prl7b1* and *Tfpi*, respectively. The intensity of *Prl7b1* staining was unchanged at GD16 and GD20 (Fig. 3A). The staining intensity of *Tfpi* in the metrial gland was significantly increased in exposed placentas at GD16 (Fig. 3B). At GD20, the MNP exposure effect was not significant although a sex-related effect on *Tfpi* intensity was detected (Fig. 3B). However, the relative distance of *Tfpi*+ cell migration from the junctional zone was significantly decreased in exposed placentas at both GD16 and GD20 (Fig. 3C). The significant interaction effect at GD20 demonstrates that this effect was more prominent in female exposed placentas while male exposed placentas experienced minimal change in relative distance of *Tfpi*+ staining (Fig. 3D). These data demonstrate that MNP exposure decreased the depth of *Tfpi*+ staining in the metrial gland, suggesting that endovascular trophoblast invasion may be impaired.

### Vascular morphology of the labyrinth zone

**Figure 4:**
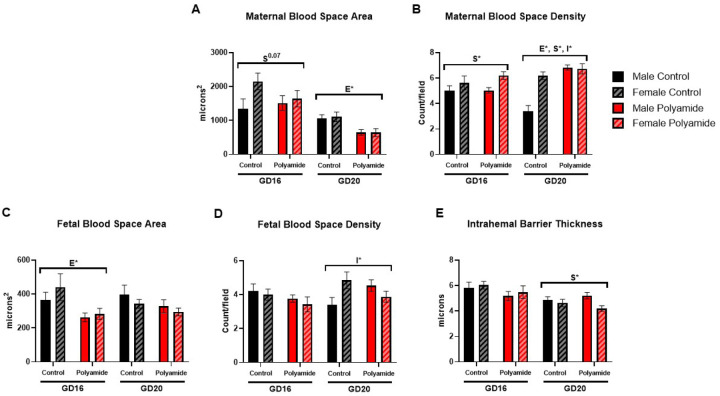
Vascular morphology within the labyrinth zone was assessed at GD16 and GD20. Maternal blood space surface area (A) and maternal blood space density (B), fetal blood space surface area (C) and fetal blood space density (D), and intrahemal barrier thickness (E) were characterized at GD16 and GD20. Data are presented as mean ± SEM. Significant exposure factor (p < 0.05) in the two-way ANOVA of age-matched placentas is denoted with E* and Ep-value when 0.05 < p < 0.1. Significant differences between male and female placentas at a given age (p < 0.05) are denoted with S* and S^p-value^ when 0.05 < p < 0.1. Significant interaction effect between male and female placentas and control and exposed placentas at a given age (p < 0.05) are denoted with I* and I^p-value^ when 0.05 < p < 0.1, n= 6–11 dams/time point/group.

Vascular development within the labyrinth zone, the site of maternal and fetal exchange, was measured in male and female placentas at GD16 and GD20. Representative images can be found in Supplemental Fig. 6. MNP exposure did not significantly change the average area of maternal blood spaces at GD16 (Fig. 4A). However, the average area of maternal blood spaces was reduced at GD20 (Fig. 4A). Maternal blood space density at GD16 was not significantly different between treatment groups (Fig. 4B). Whereas, by GD20, maternal blood space density was significantly increased in exposed placentas. This effect was more pronounced in male placentas than female placenta as evidenced by the significant interaction factor, identifying a compensation to increase maternal surface area for nutrient/waste exchange (Fig. 4B). Maternal MNP inhalation led to a decrease in average fetal blood space area at GD16 although decrease was no longer apparent by GD20 (Fig. 4C). No significant differences were found in fetal blood space count in the labyrinth zone of MNP-exposed placentas at GD16 or GD20, however the significant interaction factor suggests that exposure differentially impacted male and female placentas (Fig. 4D). Intrahemal barrier thickness, which is a measure of the diffusion distance between the maternal and fetal circulation, was not altered by MNP exposure but was found to be influenced by fetal sex (Fig. 4E). Taken together, these data suggest that gestational MNP exposure decreased the size area of the maternal and fetal blood spaces. Interestingly there are sex-related accommodations to blood space density, primarily driven by the male samples, likely increasing overall surface area for nutrient and waste exchange.

### Vascular endothelial growth factor signaling in labyrinth zone

**Figure 5:**
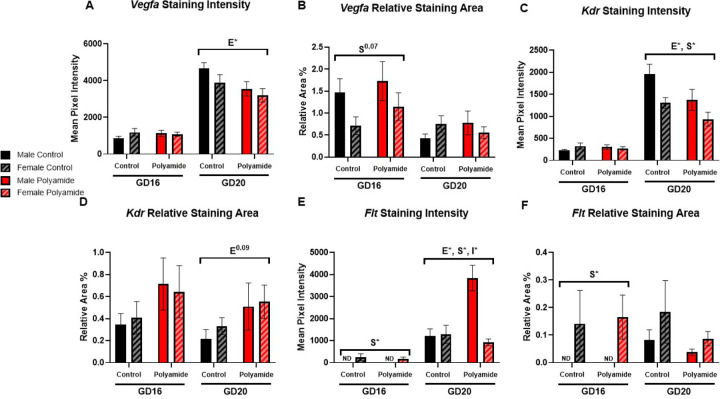
Localization of mRNA transcripts for *Vegfa*, *Kdr* and *Flt* were assessed in GD16 and GD20 placental sections. Quantification of staining intensity and relative area stained was carried out for *Vegfa* (A and B), *Kdr* (C and D) and *Flt* (E and F). Data are presented as mean ± SEM. Significant exposure factor (p < 0.05) in the two-way ANOVA of age-matched placentas is denoted with E* and Ep-value when 0.05 < p < 0.1. Significant differences between male and female placentas at a given age (p < 0.05) are denoted with S* and S^p-value^ when 0.05 < p < 0.1. Significant interaction effect between male and female placentas and control and exposed placentas at a given age (p < 0.05) are denoted with I* and I^p-value^ when 0.05 < p < 0.1, n= 6–11 dams/time point/group.

GD16 and GD20 placental sections were stained for *Vegfa*, *Kdr*, and *Flt* transcripts to assess expression of angiogenic (*Vegfa* and *Kdr*) and antiangiogenic (*Flt*) factors in the labyrinth zone. Representative images for each exposure group, age, and placental sex can be found in Supplemental Figs. 7, 8, and 9 for *Vegfa*, *Kdr*, and *Flt*, respectively. Pixel intensity for *Vegfa* was not affected in MNP-exposed placentas at GD16, but decreased in MNP-exposed placentas at GD20, independent of sex (Fig. 5A). Relative area of positive *Vegfa* staining was not significantly different between exposed and control placentas at GD16 or GD20 (Fig. 5B). *Kdr* pixel intensity was not different between control and exposed groups at GD16 but was significantly decreased in exposed placentas at GD20 (Fig. 5C). Sex of the placenta influenced this outcome with control and exposed males demonstrating increased *Kdr* pixel intensity in the labyrinth zone as compared to females. MNP exposure did not change *Kdr* relative staining area at GD16 (Fig. 5D). However, by GD20 relative *Kdr* staining area was elevated in exposed placentas however this was not to significance (p = 0.09) (Fig. 5D). *Flt* staining intensity in the labyrinth zone was not changed by exposure at GD16 however, exposure significantly increased *Flt* staining intensity by GD20 (Fig. 5E). For the measure, the interaction effect was significant, highlighting that males were more impacted and demonstrating that the effects of maternal MNP exposure on placental *Flt1* are heavily influenced by sex (Fig. 5E). No significant differences in relative *Flt* staining were observed at GD16 or GD20 (Fig. 5F). These data suggest that most MNP-induced changes to the VEGF signaling pathway occur after GD16 and that males are susceptible to increased placental antiangiogenic signaling after maternal MNP exposure.

### Angiopoietin signaling in the labyrinth zone

**Figure 6:**
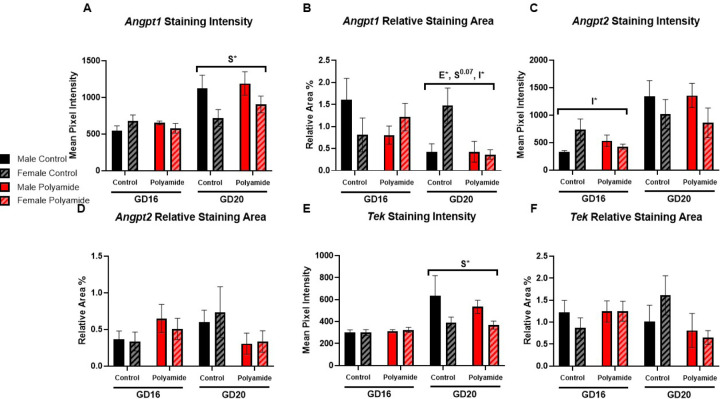
Localization of mRNA transcripts for *Angpt1*, *Angpt2* and *Tek* were assessed in GD16 and GD20 placental sections. Quantification of staining intensity and relative area stained was carried out for *Angpt1* (A and B), *Angpt2* (C and D) and *Tek* (E and F). Data are presented as mean ± SEM. Significant exposure factor (p < 0.05) in the two-way ANOVA of age-matched placentas is denoted with E* and E^p-value^ when 0.05 < p < 0.1. Significant differences between male and female placentas at a given age (p < 0.05) are denoted with S* and S^p-value^ when 0.05 < p < 0.1. Significant interaction effect between male and female placentas and control and exposed placentas at a given age (p < 0.05) are denoted with I* and I^p-value^ when 0.05 < p < 0.1, n= 6–11 dams/time point.

GD16 and GD20 placental sections were stained for *Angpt1*, *Angpt2*, and *Tek* transcripts to assess expression of vascular stabilization (Angpt1) and disruption (Angpt2) factors, as well as their primary receptor (*Tek*) in the labyrinth zone. Representative images for each exposure group, age, and placental sex can be found in Supplemental Fig. 10, 11, and 12 for *Angpt1*, *Angpt2*, and *Tek*, respectively. Pixel intensity for *Angpt1* was not affected in exposed placentas at GD16 or GD20 (Fig. 6A). Relative area of *Angpt1* staining was not significantly different between exposed and control placentas at GD16, however, the relative area stained for *Angpt1* was significantly reduced at GD20 (Fig. 6B). The significant interaction affect suggests that relative area of *Angpt1* staining in female and male exposed placentas were differentially impact by exposure (Fig. 6B). *Angpt2* pixel staining intensity was unchanged in exposed placentas at GD16, although the interaction effect was significant, suggesting that *Angpt2* expression after maternal MNP exposure are dependent on sex at GD16 (Fig. 6C). By GD20, there were no apparent changes in *Angpt2* staining intensity (Fig. 6C). MNP exposure did not change *Angpt2* relative staining area at GD16 or GD20 (Fig. 6D). Mean pixel intensity for *Tek* in the placenta was not changed by exposure at GD16 or GD20 (Fig. 5E). However, sex did influence this outcome with control and exposed male demonstrating higher pixel intensity than female (Fig. 5E). No significant differences in *Tek* relative staining area were observed at GD16 or GD20 (Fig. 5F). These data suggest that MNP exposure generates modest changes to angiopoietin signaling at GD20 and that sex plays a role in the responses of the male and female placenta. Further studies are needed to characterize how MNPs influence overall angiopoietin signaling specific to fetal sex and stage of placental development.

### α-SMA protein expression in the labyrinth zone

**Figure 7:**
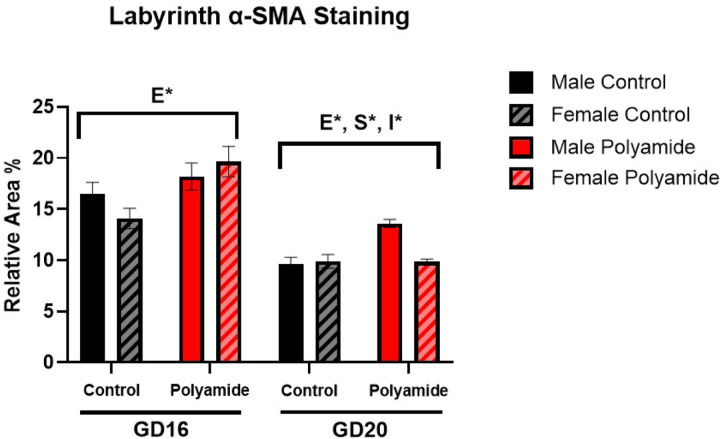
Localization of α-SMA in the labyrinth zone of the placental is shown at GD16 (A) and GD20 (B). Quantification of relative area stained is shown (C). Data are presented as mean ± SEM. Significant exposure factor (p < 0.05) in the two-way ANOVA of age-matched placentas is denoted with E* and E^p-value^ when 0.05 < p < 0.1. Significant differences between male and female placentas at a given age (p < 0.05) are denoted with S* and S^p-value^ when 0.05 < p < 0.1. Significant interaction effect between male and female placentas and control and exposed placentas at a given age (p < 0.05) are denoted with I* and I^p-value^ when 0.05 < p < 0.1, n= 6–11 dams/time point.

Relative area of α-SMA+ expression in the labyrinth zone was measured in GD16 and GD20 placentas as a possible marker of placental pericytes (Ohlsson et al. 1999; Singh et al. 2007). Representative images of α-SMA+ staining in the labyrinth of GD16 and GD20 placentas are shown in Supplemental Fig. 13. At GD16, the relative area of α-SMA+ staining in MNP exposed placentas was increased (Fig. 7). At GD20, the increase in α-SMA+ staining persisted in MNP exposed placentas (Fig. 7). However, the sex factor and interaction factor were significant, demonstrating that male α-SMA+ staining was increased in male MNP exposed placentas but not female exposed placentas (Fig. 7). Overall, MNP exposure elevated α-SMA+ staining in the labyrinth zone in an age and sex-dependent which may indicate a change in placental pericytes prevalence or function.

## Discussion

This investigation is the first to assess the effects of maternal MNP inhalation on placentation within the rat. Our data showed that MNP reduced spiral artery remodeling in the uterus. This may decrease the influx of maternal blood into the placenta although this could only be determined through direct *in vivo* measurement. We found that MNP inhalation during pregnancy alters morphology of the labyrinth zone of the placenta, which is the site of maternal-fetal exchange. Lastly, our identified changes in mRNA expression of angiogenic and antiangiogenic factors in the placenta after MNP exposure may contribute to the observed structural changes in the labyrinth zone. These findings highlight that MNP inhalation adversely impacts placental development and function which may result in detrimental effects to the maturing fetus. These studies align with others that demonstrate that MNPs influence trophoblast viability and migration, although we did not assess cell death pathways in the sampled tissue (Chen et al. 2025; Jia et al. 2025; Wan et al. 2024a). Given that multiple studies have demonstrated the ability of MNPs to deposit in placental tissue (Fournier et al. 2020; Garcia et al. 2024; Ragusa et al. 2022a) and cross the placental barrier (Dusza et al. 2022; Fournier et al. 2020; Moreno et al. 2024) in rodents and humans, more studies are needed to elucidate the activity of MNPs in placental tissue.

In our study, we characterized how MNP exposure may alter development of placental structures. While MNP exposure generated no overt pathologies in the placenta, placental weights were elevated and grams of fetal weight produced per gram of placental weight was decreased in late pregnancy. Inhalation of concentrated PM_2.5_ has previously been shown to decrease placental weight rather than increase it, as in our study (Li et al. 2022; Tao et al. 2022). Similar to our study, PM_2.5_ generated no significant changes in total placental area despite an observed change in weight (Tao et al. 2022). These data demonstrate that the placenta is a target of maternal MNP inhalation and that the effects may differ from other particle types.

Few studies have investigated how particle inhalation, including MNPs, affect maternal spiral artery remodeling in the rodent. We found MNP exposure resulted in increased prevalence of α-SMA around maternal vessels and decreased relative distance of *Tfpi* staining in the metrial gland. These data are suggestive of impaired endovascular trophoblast migration and remodeling of maternal vessels. Similarly, concentrated PM_2.5_ has been shown to increase α-SMA staining in the metrial gland of the mouse placenta (Tao et al. 2022). These data suggest that particle inhalation overall may reduce the perfusion of placental tissue. Such reduced perfusion may cause placental dysfunction as the placenta is challenged with providing nutrients to the fetus while sustaining its own nutrient demands.

Maternal MNP exposure generated structural changes to the labyrinth zone of the placenta, potentially influencing the ability of the placenta to facilitate maternal and fetal exchange. Namely, MNP exposure decreased fetal and maternal blood space size at GD16 and GD20 respectively. Increases in blood space number at GD20 may accommodate for the decrease in blood space size but how these changes influence placental hemodynamics have yet to be determined. Future studies should consider addressing cumulative maternal and fetal blood space volume in addition to the parameters assessed in this study. Other studies have shown that diesel exhaust ultrafine particle exposure increases the size of maternal and fetal blood spaces according to fetal sex (Behlen et al. 2021) although another group found that fetal capillary volume was decreased after maternal diesel exhaust exposure (Valentino et al. 2016). PM_2.5_ has been shown to increase the relative area of blood spaces in the labyrinth zone although the distinction between maternal and fetal blood spaces was not specified (Li et al. 2022). In contrast, others have found that this exposure decreased maternal and fetal blood space area (Veras et al. 2008; Yue et al. 2019). Cigarette smoke has been shown to generate similar changes with decreased size of fetal blood spaces (Chen and Yao 2017). Overall, maternal MNP inhalation influences the structures necessary for maternal-fetal exchange as demonstrated in our study.

Interestingly in our present study, we also identified a modification of α-SMA+ staining within the labyrinth zone associated with MNP exposure during gestation. This staining was associated with vascular development and placental pericytes, which have been vastly understudied in placental biology (Garcia-Gonzalez et al. 2010; Harris et al. 2021; Li et al. 2021; Moreau et al. 2014; Nadeau and Charron 2014; Natale et al. 2020; Natale et al. 2018; Ohlsson et al. 1999; Singh et al. 2007; Zhou et al. 2021). Confirmation of the α-SMA+ cell type with another marker, serial block-face scanning electron microscopy, and transmission electron microscopy would clarify how this specific cell type is potentially affected by MNP exposure. Future studies assessing how MNPs affect pericyte-fetal capillary interaction may further elucidate how MNPs influence maternal-fetal exchange.

In MNP-exposed placentas, we identified decreased mRNA expression of angiogenic factors and increased mRNA expression of anti-angiogenic factors that regulate development of the labyrinth zone. More specifically, MNP exposure resulted in reduced *Vegfa* and *Kdr* expression and *Angpt1* relative staining area while *Flt* expression was elevated. It is possible that the modulations in the relative mRNA staining area are a compensation for the measured decrease in mRNA expression, however this requires further investigation. Similar effects have been observed in models of preeclampsia (Gilbert et al. 2007; Gilbert et al. 2012; Natale et al. 2018). Concentrated PM_2.5_ has generated similar results in whole placental lysates although FLK, coded by the gene *Kdr*, was increased by exposure (Tao et al. 2022). However, another study investigating the effects of PM_2.5_ on the placenta found no significant change in *Flt* mRNA expression (Li et al. 2022). Maternal exposure generated reductions in *Vegfa* and *Angpt1* while no change was observed in *Flt* expression after cigarette smoke exposure (Chen and Yao 2017). These studies reflect the propensity MNPs to modulate angiogenic signaling in the placenta uniquely from other particles. Furthermore, these data demonstrate extensive overlaps in outcome with animal models of preeclampsia (Gilbert et al. 2007; Gilbert et al. 2012; Natale et al. 2018). This suggests that maternal MNP inhalation may similarly cause adverse fetal and offspring outcomes.

While this study is the first to address the effects of MNP inhalation in the rat placenta, careful consideration is required when interpreting these results. We used polyamide-12 MNP for this investigation of MNP toxicity. We consider this to be an environmentally relevant representative plastic, as polyamide has been identified in both environmental and biological (i.e. human tissue) samples. Polyamide-12 is not known to contain carcinogens or endocrine disrupting compounds during formulation, therefore, it may be considered low reactive as compared to other polymers. It is possible, that different effects would be observed with other polymers such as polyvinyl chloride or polyethylene. Furthermore, the plastic used in this study was a pristine plastic rather than a weathered or aged plastic. These natural processes change the physicochemical properties MNP and therefore may have an impact on interactions with biological tissues. We recognize that we utilized an occupational dose for these studies, however, we also consider these outcomes as prospective as plastic production and degradation leading to MNP formation is continuing at as exponential rate. Nonetheless, our data indicate that MNPs affect placenta in an age and sex dependent manner with varying effects between males and females at GD16 and GD20. Similar variability between sex and time point have been observed after maternal diesel exhaust and PM_2.5_ exposure, respectively (Behlen et al. 2021; Yue et al. 2019). These changes may be due to innate ability of the placenta to handle the stress of limited perfusion by modulating resource use. For example, our group has recently shown that male and female placentas have varying baseline metabolic function and varying susceptibility to the metabolic dysfunction induced after maternal nano-TiO_2_ inhalation (Seymore et al. 2025). This may in turn limit the overall health of placental tissue and the prioritization of ATP-dependent processes like endocytosis and exocytosis of molecules involved in angiogenic signaling. These data highlight the need to further explore the mechanisms of MNP-induced placental dysfunction in order to clarify the potential impacts on offspring health.

## Conclusion

The findings presented in this study reveal major implications for the developmental toxicity of MNP as it pertains to placental morphology and maturation. The environmental burden of this pervasive toxicant continues to rise exponentially. The overwhelming evidence of MNP exposure in pregnant populations shows that the interaction of MNPs with placental tissue is imminent and growing in modern society. This investigation revealed that maternal MNPs impair maternal spiral artery invasion and decrease placental surface area for maternal-fetal exchange in a sex-dependent manner. Furthermore, MNP inhalation introduced an imbalance of angiogenic and anti-angiogenic signaling to the placenta that would perpetuate a low surface area for maternal-fetal exchange. These effects on the placenta are unique from other models of particle exposure and mirror placental pathologies included preeclampsia. For this reason, inhalation of MNPs may facilitate development of a suboptimal intrauterine environment, negatively impacting fetal health. However, much remains unknown about the direct and indirect action of MNPs in placental function and development.

## Supplementary Material

Supplementary Files

This is a list of supplementary files associated with this preprint. Click to download.

- SupplementalFigures.pptx
- SupplementalTablesandFigsLegends.docx

## Figures and Tables

**Table 1:** The number of placentas from individual dams with a particular score of DAB staining circumscription around metrial gland vessels is shown. Significance was assessed via 2-way ANOVA with Sidak’s post-hoc test after transformation of data through ordered rank assignment.

Score		1	2	3	4	5	Total n in group	Median	*%* Score ≥ 3
GD16 Control	Male (n)	1	3	1	1	1	7	2	43%
	Female (n)	1	1	2	3	0	7	3	71%
GD16 Polyamide	Male (n)	3	1	3	1	2	10	3	60%
	Female (n)	4	1	0	3	2	10	3	50%
GD20 Control	Male (n)	4	4	2	1	0	11	2	27%
	Female (n)	2	0	3	2	1	8	3	75%
GD20 Polyamide	Male (n)	0	0	3	2	2	7	**4** *	100%
	Female (n)	0	0	0	6	2	8	**4** *	100%
2-way ANOVA								E*,S*	

* denotes significance (p < 0.05) when compared to sex-matched controls, E* significant exposure factor and S* denotes significant sex factor in the 2-way ANOVA results, n = 7–11/group.

## Data Availability

Data will be made available upon request.
